# FoldHSphere: deep hyperspherical embeddings for protein fold recognition

**DOI:** 10.1186/s12859-021-04419-7

**Published:** 2021-10-12

**Authors:** Amelia Villegas-Morcillo, Victoria Sanchez, Angel M. Gomez

**Affiliations:** Department of Signal Theory, Telematics and Communications, University of Granada, Periodista Daniel Saucedo Aranda, 18071 Granada, Spain

**Keywords:** Protein fold recognition, Deep neural networks, Residual convolutions, Embedding learning, Hyperspherical space, Thomson problem

## Abstract

**Background:**

Current state-of-the-art deep learning approaches for protein fold recognition learn protein embeddings that improve prediction performance at the fold level. However, there still exists aperformance gap at the fold level and the (relatively easier) family level, suggesting that it might be possible to learn an embedding space that better represents the protein folds.

**Results:**

In this paper, we propose the FoldHSphere method to learn a better fold embedding space through a two-stage training procedure. We first obtain prototype vectors for each fold class that are maximally separated in hyperspherical space. We then train a neural network by minimizing the angular large margin cosine loss to learn protein embeddings clustered around the corresponding hyperspherical fold prototypes. Our network architectures, ResCNN-GRU and ResCNN-BGRU, process the input protein sequences by applying several residual-convolutional blocks followed by a gated recurrent unit-based recurrent layer. Evaluation results on the LINDAHL dataset indicate that the use of our hyperspherical embeddings effectively bridges the performance gap at the family and fold levels. Furthermore, our FoldHSpherePro ensemble method yields an accuracy of 81.3% at the fold level, outperforming all the state-of-the-art methods.

**Conclusions:**

Our methodology is efficient in learning discriminative and fold-representative embeddings for the protein domains. The proposed hyperspherical embeddings are effective at identifying the protein fold class by pairwise comparison, even when amino acid sequence similarities are low.

**Supplementary Information:**

The online version contains supplementary material available at 10.1186/s12859-021-04419-7.

## Background

Protein structure prediction given the amino acid sequence is a challenging problem in structural bioinformatics. One of the key steps in the template-based modelling (TBM) of protein structures is the recognition of the protein fold [1–5]. The goal is to predict the fold type of a protein domain by comparison with template structures from the Protein Data Bank (PDB) [6]. Solved structure domains from the PDB are classified into several levels according to structural and sequence similarities in databases as SCOP [7, 8] and CATH [9]. The objective here is to identify proteins sharing the same *fold class*—with similar arrangement of structural elements but differing in the amino acid sequence.

Early computational approaches to recognizing proteins with similar structure and sequence (homology modelling) were based on sequence-to-sequence (BLAST [10]) or profile-to-profile (HHpred [11]) alignments, as well as Markov random fields (MRFAlign [12]). In addition, threading methods aim to recognize distant-homologous proteins with low similarity in sequence by using structural properties instead. These methods include RAPTOR [13], BoostThreader [14], SPARKS-X [15], conditional random field-based CNFpred [16] and [17], and more recently the EigenTHREADER [18] and CEthreader [19] methods, which use predicted contact map information.

In general, the protein fold recognition methods as the ones described above are derived from the template-based structure prediction problem. Unlike these, in the taxonomy-based fold classification approaches [20] the protein sequences are directly mapped into fold classes. To this end, machine learning approaches such as FP-Pred [21], ACCFold [22], TAXFOLD [23–25], HMMFold [26], ProFold [27], and DKELM-LDA [28], as well as the deep learning methods Conv-SXGbg-DeepFold [29] and DeepFrag-k [30], have been proposed to successfully classify into a pre-defined group of SCOP fold classes. However, the evaluated folds comprise a small set including only those folds with a higher amount of protein domains (27 or 30 folds), in contrast to the more than 1000 existing fold classes in the SCOP database.

Several machine learning algorithms have been also introduced for the protein fold recognition task [31]. First attempts treated the task as a binary classification problem to decide whether two protein domains belonged to the same fold. Different techniques were applied here, such as support vector machines (FOLDpro [32]), random forests (RF-Fold [33]) and neural networks (DN-Fold [34]). Moreover, ensemble methods enhance the recognition performance by combining multiple protein feature representations and prediction techniques. Examples are TA-Fold [35] and the multi-view learning ensemble frameworks MT-fold [36], EMfold [37] and MLDH-Fold [38]. On the other hand, the learning to rank methods, such as Fold-LTR-TCP [39], FoldRec-C2C [40], and ProtFold-DFG [41], treat the problem as an information retrieval task and try to learn the relationship among proteins in the datasets.

Furthermore, deep learning-based methods have been recently proposed to identify the protein fold, such as DeepSF [42], DeepFR [43], DeepSVM-Fold [44], MotifCNN-fold [45], SelfAT-Fold [46], VGGfold [47], and CNN-BGRU [48]. In these methods, a supervised neural network model is trained to classify the input protein domain into one of the possible fold classes. From the trained model, a fold-related embedding representation is extracted, which is then used to measure the similarity between each two protein domains. In this context, the learned embeddings constitute a *d*-dimensional space in which we can map high-dimensional protein representations such as evolutionary profiles [48] ($L\times 20$, where *L* is the protein sequence length) or contact maps [43] ($L\times L$). Moreover, these embeddings capture the fold information during training by placing inputs from the same fold close together in the embedding space. The model architecture for protein fold recognition usually contains a convolutional neural network (CNN) alone or in combination with recurrent layers—long-short term memory (LSTM) [49] or gated recurrent unit (GRU) [50] cells—or self-attention layers [51]. Hence, in preceding works, most of the effort has been put into improving the neural network architectures and making them suitable to process different protein representations, such as predicted contact maps, evolutionary profiles or predicted secondary structure elements. In this work, we propose two architectures formed by several blocks of residual-convolutions [52] and a recurrent layer, which we name ResCNN-GRU and ResCNN-BGRU. Here, the suffix ‘BGRU’ refers to bidirectional GRU, while ‘GRU’ indicates the use of a unidirectional GRU. These two architectures are derived from our previous CNN-GRU and CNN-BGRU models [48], and can also process arbitrary length protein sequences represented by residue-level features.

However, unlike previous deep learning approaches, our main interest here is to improve the fold-related embedding vectors by modifying the neural network optimization criterion. While the softmax cross-entropy loss is commonly used for multi-class classification problems, it lacks sufficient discriminative power for classification [53–55]. In this regard, modifications on the loss function have been introduced, leading to improved functions such as the center loss [53], the large margin softmax (L-Softmax) loss [54], and the angular softmax (A-Softmax) loss [55]. Thus, in this work, we propose to minimize an angular-based loss function, namely the *large margin cosine loss* (LMCL) [56]. LMCL removes any vector norm dependencies by normalizing the input embedding and class weight vectors in the classification layer and therefore distributes them angularly on a high-dimensional sphere—or *hypersphere*. The function also introduces a class boundary margin to enlarge the inter-class angular separation while reducing the intra-class separation for embeddings within the same fold class.

We further improve the training of our neural network model by minimizing the LMCL with a fixed weight matrix in the last classification layer. Such a matrix contains a pre-defined set fold class vectors—*hyperspherical prototypes*—that are maximally separated on the surface of a hypersphere. To ensure maximum angular separation between prototypes, we draw inspiration from the well-known Thomson problem [57]. Its goal is to determine the minimum energy configuration of *K* charged particles on the surface of a unit sphere. By minimizing a Thomson-based loss function, extended to a hypersphere of arbitrary number of dimensions, we optimize the angular distribution of our prototype vectors. Here we pre-train the prototype matrix separately and keep it fixed during the optimization of our neural network model. It must be noted that, unlike conventional transfer learning procedures in which the last layers of the network are fine-tuned, we pre-define the output embedding space given by a set of fold prototypes representing the cluster centroids for each fold class [58]. In this way, during training, the model is forced to learn protein embeddings clustered around the corresponding hyperspherical fold prototypes.

**Fig. 1:**
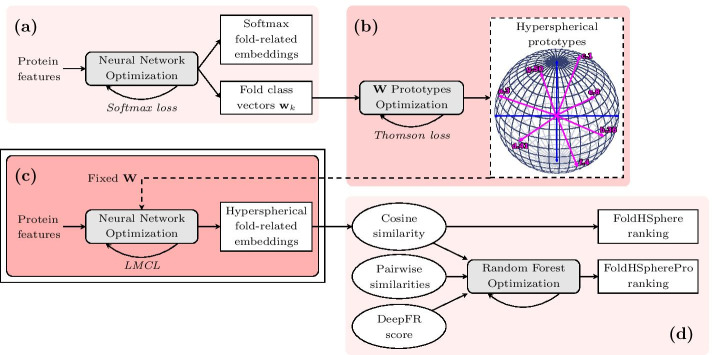
Overview of the FoldHSphere approach for protein fold recognition. In the first stage **a** we train a neural network model to map the protein domains into *K* fold classes using the softmax cross-entropy as loss function. From this trained model, we extract fold class weight vectors $\mathbf {w}_k, k=1,\dots ,K$ learned in the last classification layer. **b** We then optimize the position of the $\mathbf {w}_k$ vectors by our proposed Thomson-based loss, so that they are maximally separated in the angular space. **c** The resulting hyperspherical prototypes are used as a fixed non-trainable classification matrix $\mathbf {W}$ in the last layer of the neural network model, which is trained again, but now minimizing the LMCL. The final hyperspherical embeddings are extracted from the fully-connected part of this model. **d** Finally, the cosine similarity is computed between each two embeddings and a template ranking is performed for each query protein domain (FoldHSphere method). Moreover, template ranking is further improved by using enhanced scores provided by a random forest model trained with additional similarity measures as inputs (FoldHSpherePro method)

In summary, our main contribution is a training procedure that provides hyperspherical protein embeddings, learned by minimizing the angular LMCL around pre-defined prototypes for the fold classes in a hyperspherical space. We obtain these embeddings by training the ResCNN-GRU and ResCNN-BGRU architectures that are effective at processing arbitrary length protein sequences. An overview of our approach is depicted in Fig. 1. Our proposed methods, named FoldHSphere and FoldHSpherePro, significantly advance the state-of-the-art performance on well-known benchmark datasets.

## Materials and methods

### Datasets

The datasets were obtained from the public protein databases SCOP [7] and the extended SCOPe [8]. These databases contain a hierarchical structural classification of protein domains with solved structure. From the top-down view, such hierarchical levels are *structural class, fold, superfamily* and *family*, which group protein domains with increasing sequence similarity at each level.

#### Training dataset

We trained our neural network models using the SCOPe 2.06 training set from [43]. Such a training set was obtained after filtering out protein domains having a significant sequence similarity to those in the test set. To do so, the following similarity reduction methods were executed: MMseqs2 [59] (sequence identity 25%, e-value $10^{-4}$), CD-HIT-2D [60] (sequence identity 40%) and BLAST+ [61] (e-value $10^{-4}$). The final dataset contains 16133 protein domains sharing at most 95% pairwise sequence identity, which are classified into $K=1154$ folds. For hyperparameter tuning, we performed a 5-stage cross-validation over the entire training set. Hence, we split the 16,133 protein domains into 5 groups, including domains from different families in each one (Additional file 1: S1). This prevents having proteins in the validation subsets with similar amino acid sequence to those in the corresponding training subset.

#### Benchmark datasets

We tested the effectiveness of our hyperspherical embeddings using both the well-known LINDAHL dataset [3] and the updated LINDAHL_1.75 dataset we recently proposed in [48]. The original LINDAHL dataset includes 976 domains from SCOP 1.37 covering 330 folds. Updated to SCOP 1.75, the LINDAHL_1.75 dataset contains the same number of proteins (976) but now classified into 323 folds. Protein domains within both test sets share a maximum sequence identity of 40%, as well as with respect to the training domains. Each dataset is paired and evaluated independently at three different levels—*family, superfamily* and *fold*. Thus, while the number of individual protein domains evaluated within the LINDAHL dataset are 555, 434 and 321 for the family, superfamily and fold levels, in LINDAHL_1.75 we evaluate 547, 431 and 356 domains, respectively.

### Protein residue-level feature representation

In order to represent the protein amino acid sequence with variable length *L*, we considered 45 features for each amino acid as in previous works [42, 48]. These 45 residue-level features contain the following information:

- *Amino acid encoding:* one-hot vector of size 20 representing the amino acid type.
- *Position-specific scoring matrix (PSSM):* 20 elements which contain the evolutionary profile information obtained from the multiple sequence alignment (MSA). We computed the PSSM matrix using PSI-BLAST [10] and the non-redundant database ‘nr90’ for sequence homology searching.
- *Secondary structure:* one-hot vector of size 3 encoding the helix, strand and loop secondary structure elements. To predict the secondary structure we used the SSpro method from the SCRATCH suite [62].
- *Solvent accessibility:* one-hot vector of size 2 encoding the exposed and buried states. Similar to before, we used the ACCpro method from SCRATCH to predict the solvent accessibility states.

These $L\times 45$ features are used as input to our neural network models, which are trained to predict the fold class for each protein domain.

### Residual-convolutional and recurrent neural network

**Fig. 2:**
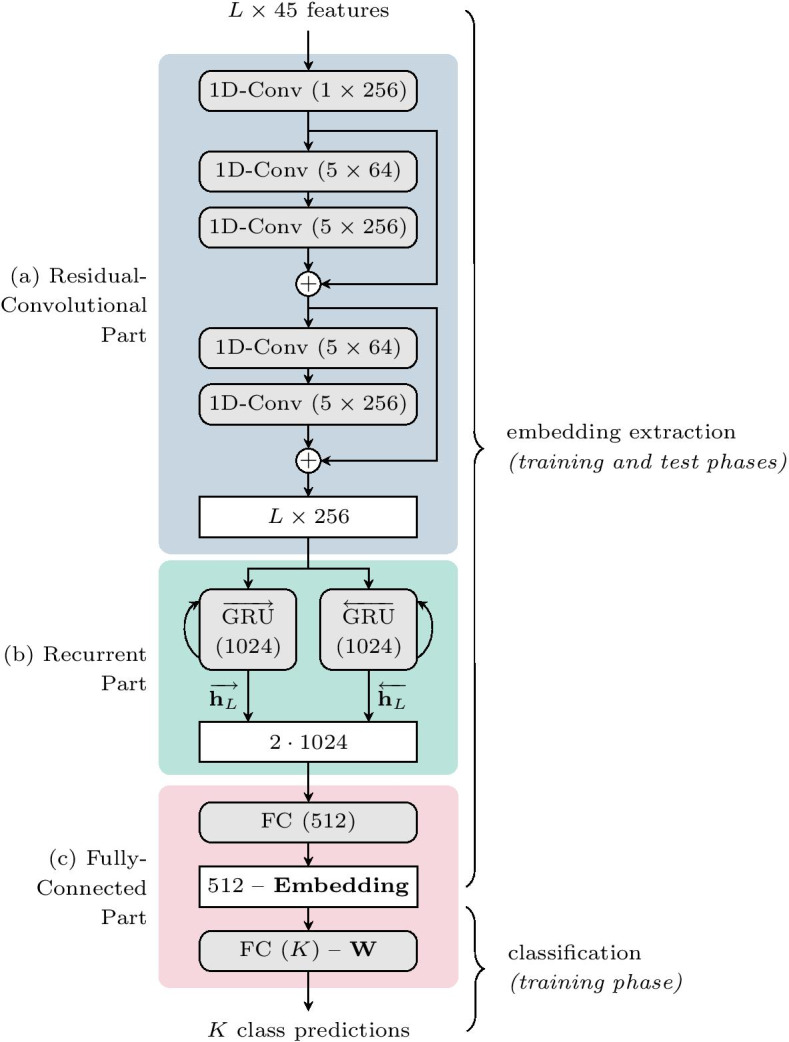
The proposed ResCNN-BGRU neural network model for fold-related embedding learning through protein fold classification. The model architecture contains three differentiated parts. The residual-convolutional network **a** processes the input $L\times 45$ residue-level features and consists of two residual blocks with two 1D-convolutional layers each. Its output is passed through a bidirectional layer of gated recurrent units (**b**) to obtain a fixed size representation of the input domain, which is further processed by two fully-connected layers (**c**). The first FC layer learns a 512-dimensional embedding vector for each input, while the second one learns a class weight matrix $\mathbf {W}$ to perform the classification into *K* fold classes. The ResCNN-GRU model is identical but using a unidirectional GRU layer instead

In this study, we improve our previously proposed neural network models, CNN-GRU and CNN-BGRU [48], with blocks of residual convolutions [52]. As a result, the model architecture is formed by three main parts, as depicted in Fig. 2: residual-convolutional (ResCNN), recurrent (RNN) and fully-connected (FC). We named these new models as ResCNN-GRU and ResCNN-BGRU, depending on the use of unidirectional or bidirectional layers of gated recurrent units (GRU) in the recurrent part.

#### Residual-convolutional part

The convolutional neural network (CNN) aims to capture the local context of each residue in the protein domain and discover short-term patterns within the amino acid sequence. At each CNN layer, we apply a 1D-convolution operation along the sequence dimension, with several convolutional filters of specific length to be learned. Considering an input of size $L\times 45$, the output of each 1D-convolutional layer is of size $L\times N_l$, where $N_l$ is the number of learned filters in the *l*-th layer. In our model, the 1D-convolutional layers are grouped into residual blocks [52]. The output $\mathcal {R}(x_b, \mathcal {W}_b)$ of each residual block is combined with its input $x_b$ as $x_{b+1} = x_b + \mathcal {R}(x_b, \mathcal {W}_b)$, where $\mathcal {W}_b$ are the weights and biases associated to the *b*-th residual block, and $\mathcal {R}(\cdot )$ is the mapping function performed by the block.

Figure 2a presents the ResCNN part of our model. We first apply an initial 1D-convolution to transform the $L\times 45$ input features into $L\times 256$ outputs by using 256 filters of length 1. These are then processed by two residual blocks, each one formed by two layers with 64 and 256 filters of length 5. After each convolution, ReLU activation and Batch-Normalization [63] are applied.

#### Recurrent part

The purpose of the recurrent neural network (RNN) is to exploit long-distance relations through all the amino acid sequence and generate a summary of the whole protein domain at its output. Here, the $L\times 256$ outputs from the ResCNN are fed into a gated recurrent unit (GRU) [50] based layer with 1024 state units.

As shown in Fig. 2b, instead of saving all the $L\times 1024$ states of the GRU, we only consider the last state ($\overrightarrow{\mathbf {h}_L}$) as a summary vector of 1024 elements. In this way, our model architecture can process amino acid sequences of arbitrary length and extract a fixed-size vector representing the whole protein domain. We refer to this model as ResCNN-GRU. An alternative architecture is that based on a bidirectional GRU [64] which also processes the sequence in reverse order. In such a case, last states from both forward ($\overrightarrow{\mathbf {h}_L}$) and backward ($\overleftarrow{\mathbf {h}_L}$) GRU layers are concatenated into a vector of 2048 elements. We denote this model as ResCNN-BGRU.

#### Fully-connected part

Finally, the fully-connected (FC) part combines the recurrent output to create a fold-related embedding for the whole protein domain, which is then used to perform a preliminary fold classification. The classification step guides the model during training to learn a meaningful embedding space, which is related to the protein folds. Then, these learned embeddings are used for pairwise fold recognition in the test phase.

In particular, the FC part (Fig. 2c) consists of two dense layers. The first one, with 512 units, is used to learn a nonlinear combination of the GRU output vector (1024 or 2048 for the unidirectional and bidirectional architectures, respectively) which shapes the fold-related embedding. As nonlinearity, both the sigmoid and the hyperbolic tangent (tanh) activation functions have been tested in our experiments. The last layer performs a linear classification of the 512-dimensional embeddings using *K* output units. Here, *K* is the number of fold classes in which the input proteins are classified during training. In the following subsections we detail how this last classification layer can be modified to learn more discriminative embedding vectors by distributing the fold class vectors in hyperspherical space.

### Neural network model optimization

We trained our neural network models with mini-batches of 64 protein domains. To process variable-length sequences, we applied zero-padding to the maximum length within each mini-batch. After the GRU layer, we kept the last state vector of each domain sample before the zero-padding, which corresponds to the last amino acid residue of each domain in the mini-batch. In the bidirectional GRU, the same GRU layers are used but the amino acid sequences were first reversed for the backward layer, so the last state (before zero-padding) corresponds to the first residue of each domain. The optimization process was performed in two different stages by comparing the model predictions with the true fold classes (ground truth). In the first one (Fig. 1a), we optimized the models by minimizing the well-known softmax cross-entropy loss, while in the second stage (Fig. 1c) we used the large margin cosine loss (LMCL) [56], which is a normalized and margin discriminative version of the softmax loss. In this case, we also used a fixed (i.e. non-trainable) weight matrix in the classification layer ($\mathbf {W}$ in Fig. 2c) which maximally separates fold class vectors in hyperspherical space (Fig. 1b). We used the Adam optimizer [65] with an initial learning rate of $10^{-3}$, which we reduced by a factor of 10 at epoch number 40, whereas the whole optimization process was completed in 80 epochs. In order to prevent overfitting to the most populated fold classes, we applied $L_2$ penalty with a small weight decay of $5\times 10^{-4}$ and dropout [66] with a drop probability of 0.2 in the convolutional and the first FC layers.

### Large margin cosine loss

The softmax cross-entropy loss (softmax loss for simplicity) is one of the most common loss functions for multi-class classification problems. It is defined as:

1 $$\begin{aligned} L_{softmax} = - \frac{1}{N} \sum _{i=1}^N \log p_i = - \frac{1}{N} \sum _{i=1}^N \log \frac{ e^{f_{y_i}} }{ \sum _{k=1}^K e^{f_k} }, \end{aligned}$$

where $p_i$ is the posterior probability of the $\mathbf {x}_i$ embedding sample being classified into its ground-truth class $y_i$, *N* is the number of training samples in the mini-batch ($i={1, \dots , N}$), *K* is the number of classes ($k={1, \dots , K}$), and $f_k$ is the output of the last linear classification layer with weight matrix $\mathbf {W} \in \mathbb {R}^{K\times d}$ (the bias is set to zero for simplicity). For each input $\mathbf {x}_i$, the output corresponding to class *k* is computed as:

2 $$\begin{aligned} f_k = \mathbf {w}_k^T \mathbf {x}_i = \left\| \mathbf {w}_k \right\| \left\| \mathbf {x}_i \right\| \cos (\theta _{k,i}), \end{aligned}$$

with $\theta _{k,i}$ being the angle between the vectors $\mathbf {w}_k$ and $\mathbf {x}_i$. If we enforce that $\left\| \mathbf {w}_k \right\| = 1$ through $L_2$ normalization, and $\left\| \mathbf {x}_i \right\| = s$ by using a tunable scale hyperparameter, the posterior probability only depends on the cosine of the angle $\theta _{k,i}$. This results in the normalized softmax loss (NSL), defined as:

3 $$\begin{aligned} L_{ns} = - \frac{1}{N} \sum _{i=1}^N \log \frac{ e^{s \cos (\theta _{y_i,i}) } }{ \sum _{k=1}^K e^{s \cos (\theta _{k,i})} }. \end{aligned}$$

The feature embeddings learned by NSL are angularly distributed, but they are not necessarily more discriminative than the ones learned by softmax loss. In order to control the classification boundaries, two variants of the NSL, the angular softmax (A-Softmax) loss [55] and the large margin cosine loss (LMCL) [56], introduce a margin hyperparameter ($m \ge 0$). The decision margin in LMCL is defined in cosine space rather than in angle space, which proved to be more beneficial when learning the classification boundaries [56]. This is therefore the loss function we adopted to optimize our neural network models, and is formally defined as:

4 $$\begin{aligned} L_{lmc} = - \frac{1}{N} \sum _{i=1}^N \log \frac{ e^{s (\cos (\theta _{y_i,i}) - m)} }{ e^{s (\cos (\theta _{y_i,i}) - m)} + \sum _{k\ne y_i} e^{s \cos (\theta _{k,i})} }, \end{aligned}$$

subject to $\cos (\theta _{k,i}) = \hat{\mathbf {w}}_k^T \hat{\mathbf {x}}_i$, where $\hat{\mathbf {w}}_k$ and $\hat{\mathbf {x}}_i$ are the $L_2$ normalized vectors ($\hat{\mathbf {w}}_k = \mathbf {w}_k / \left\| \mathbf {w}_k \right\|$ and $\hat{\mathbf {x}}_i = \mathbf {x}_i / \left\| \mathbf {x}_i \right\|$).

As stated in the original paper [56], by $L_2$-normalizing the embedding vectors $\mathbf {x}_i$, we enforce them to be distributed on the surface of a *d*-dimensional hypersphere. Thus, the scaling hyperparameter *s* controls the radius of such hypersphere and its value increases with the number of classes. The margin hyperparameter *m* relates to the capacity of learning more discriminative embeddings. Possible values are in the range $m \in [ 0, \frac{K}{K-1} )$, although high values close to the upper-bound could cause failures in convergence. Having this in mind, we tuned the scale *s* and margin *m* hyperparameters for each neural network model through cross-validation.

### Thomson-derived hyperspherical prototypes

We hypothesize that by providing a non-trainable matrix $\mathbf {W} \in \mathbb {R}^{K\times d}$ to the classification layer we can ease the training process. Such matrix contains *K* pre-defined prototype vectors representing each fold class, $\mathbf {W} = \{ \mathbf {w}_1, \dots , \mathbf {w}_K \}$. Thus, we can shape the embedding space to be representative of the protein folds, and so extract more meaningful fold-related embeddings for each protein during the training stage (Fig. 1c). The use of such prototype networks was first proposed in [58].

#### Optimal distribution of prototypes

We argue that the optimal configuration of the *K* prototype vectors is that which provides maximal separation in the angular space. This can be achieved by placing the *K* points equidistant on the surface of a *d*-dimensional hypersphere, so $\mathbf {w}_k \in \mathbb {S}^{d-1}$, as shown in Fig. 1b. The Thomson problem [57] addresses this by studying the distribution of *K* charged particles on the surface of a unit 3D-sphere. The minimum energy configuration can be optimized by measuring the Coulomb’s law. When using simplified units for electron charges and Coulomb’s constant, the formula for a pair of electrons reduces to $E_{ij} = 1 / r_{ij}$, relying only on the distance ($r_{ij}$) between the two points.

This can be extended to points located on the surface of a hypersphere of *d* dimensions and computed for all possible pairs of points [67]. We could therefore optimize the distribution of our $\mathbf {w}_k$ prototype vectors by minimizing the generalized Thomson loss (THL), defined as:

5 $$\begin{aligned} L_{th} = \sum _{k=1}^K \sum _{j=1}^{k-1} \frac{1}{\left\| \mathbf {w}_k - \mathbf {w}_j \right\| _2^2} + \frac{\lambda }{2} \sum _{k=1}^K (\left\| \mathbf {w}_k \right\| ^2 - 1)^2. \end{aligned}$$

The hyperparameter $\lambda$ controls the weight of the norm constraint. Note that the Thomson loss uses the Euclidean distance between points, which is affected by the norm of each vector, while the cosine similarity is more adequate to measure the angular separation (independent of the norm). In order to remove the norm constraint from the loss function, we propose to directly maximize the Euclidean distance of the projected ($L_2$-normalized) vectors. Thus, we can remove the hyperparameter $\lambda$ from equation (5), obtaining the following Thomson loss (THL–*sum*):

6 $$\begin{aligned} L_{th\_sum} = \sum _{k=1}^K \sum _{j=1}^{k-1} \left\| \frac{\mathbf {w}_k}{\left\| \mathbf {w}_k \right\| } - \frac{\mathbf {w}_j}{\left\| \mathbf {w}_j \right\| } \right\| _2^{-2}. \end{aligned}$$

Alternatively, we can instead minimize the maximum cosine similarity computed for each prototype vector [58], using the following loss function (THL–*maxcos*):

7 $$\begin{aligned} L_{th\_maxcos} = \frac{1}{K} \sum _{k=1}^K \max _{j\ne k} \left( \frac{\mathbf {w}_k \cdot \mathbf {w}_j}{\left\| \mathbf {w}_k \right\| \left\| \mathbf {w}_j \right\| } \right) . \end{aligned}$$

Maximally separated prototype vectors are obtained by means of gradient descent over the proposed loss function (either THL–*sum* or THL–*maxcos*), where it must be noted that all possible pairs of points are taken to perform a single iteration step.

#### Initial prototype vectors

As initial matrix of prototypes we can consider a set of *K* Gaussian random variables of dimension *d*, $\mathbf {W}^{random}$. However, we found that the learned classification matrix from a model previously trained with the softmax cross-entropy loss (Fig. 1a), $\mathbf {W}^{softmax}$, provides better results. Unlike $\mathbf {W}^{random}$, the matrix $\mathbf {W}^{softmax}$ has been trained to classify protein domains into folds, somehow preserving the arrangement of the structural classes within the learned space. To show this, we measured the intra- and inter-structural class prototype separation, as well as the angular Fisher score (AFS) [55]. Further details can be found in Additional file 1: S2.

### Pairwise similarity scores

#### Cosine similarity measures

The FoldHSphere method (Fig. 1d) uses the hyperspherical embeddings extracted from our neural network model to compute a fold similarity measure between each pair of protein domains. Following previous works [43, 48], we used the cosine similarity between two embedding vectors $[ \mathbf {x}_i, \mathbf {x}_j ] \in \mathbb {R}^{d}$ as metric, computed as:

8 $$\begin{aligned} \cos (\mathbf {x}_i, \mathbf {x}_j) = \frac{\mathbf {x}_i \cdot \mathbf {x}_j}{\left\| \mathbf {x}_i \right\| \left\| \mathbf {x}_j \right\| }, \end{aligned}$$

which is a measure of angular separation (in the range $[-1, 1]$) and independent of the norm of each embedding vector.

#### Random forest enhanced scores

To obtain an improved fold similarity score (FoldHSpherePro in Fig. 1d), we trained a random forest (RF) model using our cosine similarity score along with the 84 pairwise similarity measures from [33, 34] and the DeepFR cosine similarity [43]. Thus, each input vector is of size 86 and corresponds to a pair of protein domains. The RF model uses this information to determine whether the domains in such a pair share the same fold class (binary classification). We trained and evaluated the RF models in a 10-stage cross-validation setting for the LINDAHL and LINDAHL_1.75 test sets independently. The random forest models used 500 decision trees each as in [43, 48].

### Evaluation

#### Three-level rank performance accuracy

As originally proposed in [3], we evaluated the test protein domains at three levels of increasing difficulty—*family, superfamily* and *fold*. At each level, we differentiated between positive and negative pairs of domains. A negative pair contains two protein domains from different fold classes, while in a positive pair both domains are from the same fold class. Each level includes all the negative pairs, while positive pairs are selected according to the SCOP hierarchy [7]. That is, the *family level* contains pairs of domains that share the same family class, and therefore the same superfamily and fold classes. At the *superfamily level*, the domains in each pair share the same superfamily class—and therefore the same fold—but not the same family. Finally, domains in positive pairs at the *fold level* only share the same fold class, but neither share the same family nor superfamily.

At each of these levels, for every individual protein domain (query) we ranked the rest of domains (templates) according to their similarity scores. These can be either cosine similarities or random forest output scores. Then, we assigned the fold class of the most similar template to the query and computed the ratio of hits—*top 1 accuracy*. We also obtained the ratio of finding the correct fold class within the 5 first-ranked templates—*top 5 accuracy*. It must be noted that, instead of using the training set as the search database, in this evaluation we aim to find template domains inside the test set itself (either LINDAHL or LINDAHL_1.75).

In order to measure the statistical significance of our top 1 and top 5 accuracy results, we also provide standard errors estimated as the standard deviation of 1000 bootstrap samples. To do so, we sampled with replacement from the set of individual protein domains that are tested at each level (555, 434 and 321 domains respectively in the LINDAHL dataset). Then, for each sampled set we selected all negative pairs and positive pairs corresponding to the specific level, and proceeded with the evaluation as before.

#### Fold-level LINDAHL cross-validation evaluation

In order to compare with some recent methods [35–41, 44–46] we also provide results on a fold-level 2-stage cross-validation setting on the LINDAHL test set [22]. Here, the 321 protein domains which form positive pairs at the fold level are separated into two subsets LE_a and LE_b, with 159 and 162 domains each. Note that the rest of domains within LINDAHL (up to 976) are not considered during this evaluation. When evaluating the protein domains in each subset (e.g. LE_a), the domains in the other subset (LE_b) act as templates for ranking. Thus, the random forest models are trained using pairs of protein domains from one subset, whereas the evaluation is performed on the other one. In this evaluation, we report the averaged performance accuracy over both cross-validation subsets.

## Results

**Fig. 3:**
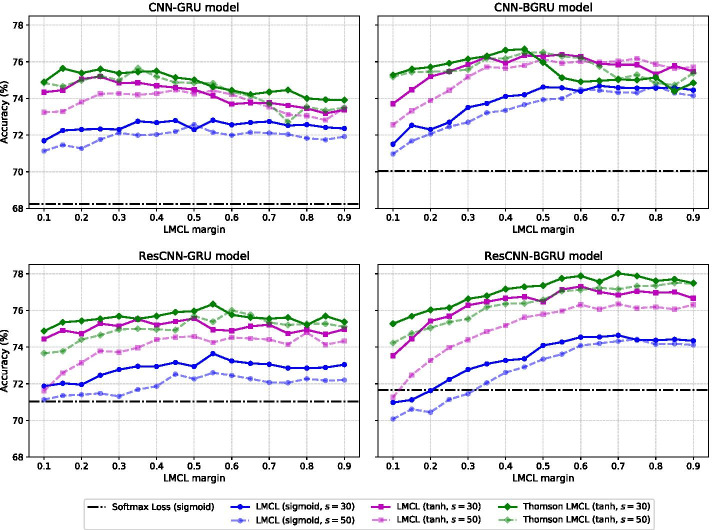
Cross-validation fold classification accuracy (%) results for different LMCL margins and scales $s=\{30, 50\}$, using the SCOPe 2.06 training set. The results are provided separately for each neural network model: CNN-GRU, CNN-BGRU, ResCNN-GRU and ResCNN-BGRU, trained using different combinations of activation function (in the embedding layer) and loss function. These are: softmax loss with sigmoid activation (dash-dotted horizontal line), LMCL with sigmoid activation (blue lines), LMCL with tanh activation (magenta lines) and Thomson LMCL with tanh activation (green lines). For the LMCL and Thomson LMCL results, solid lines and dashed lines correspond to scale values 30 and 50, respectively

### Learning fold-related embeddings with LMCL

We first assessed the performance of the different neural network models trained either with the softmax loss (1) or the LMCL (4) (see Fig. 1a), by cross-validation over the SCOPe 2.06 training set. For the softmax loss, we used the sigmoid activation in the embedding layer (first FC layer in Fig. 2c), so that we can compare with the CNN-GRU and CNN-BGRU models from [48]. Then, for each model trained with the LMCL function, we tuned the scale and margin hyperparameters through cross-validation. We considered two values for the scale $s=\{30, 50\}$ and margins in the range $m=[0.1,0.9]$. Here we tested two activation functions at the embedding layer: sigmoid as well as hyperbolic tangent (tanh). We argue that having negative and positive values ranging from $-1$ to 1 in the embedding vector (tanh activation) would better exploit the hyperspherical space than having only positive values (sigmoid activation, range [0, 1]).

**Table 1:** Optimal set of hyperparameters for the LMCL function

Model	(a) LMCL		(b) Thomson LMCL		
	Scale	Margin	Iter THL-sum	Scale	Margin
CNN-GRU	30	0.25	1130	30	0.25
CNN-BGRU	30	0.55	1172	30	0.45
ResCNN-GRU	30	0.50	1181	30	0.55
ResCNN-BGRU	30	0.60	1020	30	0.60

The scale and margin hyperparameters are provided for each neural network model and two approaches: (**a**) training the last classification layer end-to-end, (**b**) using the fixed prototype matrix by minimizing the Thomson loss THL–*sum*. We also include here the optimal iteration from the Thomson algorithm

**Table 2:** Effect of model architecture and loss function choice on FoldHSphere performance using the LINDAHL dataset

Model	Family		Superfamily		Fold	
	Top 1	Top 5	Top 1	Top 5	Top 1	Top 5
*(a) Softmax loss*						
CNN-GRU [48]	68.6 (1.94)	89.2 (1.37)	56.2 (2.34)	77.4 (1.96)	56.7 (2.82)	74.1 (2.46)
CNN-BGRU [48]	71.0 (1.92)	87.7 (1.42)	60.1 (2.30)	77.2 (2.02)	58.3 (2.83)	**78.8** (2.27)
ResCNN-GRU	72.6 (1.87)	90.3 (1.24)	59.4 (2.32)	77.0 (2.00)	58.9 (2.88)	75.1 (2.44)
ResCNN-BGRU	**76.8** (1.78)	**91.2** (1.23)	**65.0** (2.29)	**82.0** (1.84)	**59.5** (2.79)	76.6 (2.35)
*(b) LMCL*						
CNN-GRU	**76.6** (1.80)	**90.8** (1.25)	64.7 (2.21)	80.2 (1.90)	65.7 (2.69)	79.8 (2.22)
CNN-BGRU	76.2 (1.79)	89.4 (1.31)	**70.5** (2.12)	83.2 (1.80)	72.0 (2.48)	81.0 (2.21)
ResCNN-GRU	75.7 (1.77)	89.7 (1.25)	66.4 (2.29)	81.1 (1.86)	67.6 (2.63)	80.1 (2.23)
ResCNN-BGRU	75.1 (1.84)	89.5 (1.30)	69.8 (2.25)	**85.3** (1.67)	**74.1** (2.42)	**82.2** (2.12)
*(c) Thomson LMCL*						
CNN-GRU	**80.0** (1.73)	90.6 (1.24)	66.8 (2.23)	80.2 (1.94)	66.0 (2.62)	80.1 (2.22)
CNN-BGRU	77.5 (1.75)	**91.7** (1.19)	69.8 (2.09)	85.3 (1.64)	72.6 (2.48)	82.2 (2.14)
ResCNN-GRU	76.9 (1.78)	89.5 (1.28)	69.1 (2.20)	82.9 (1.77)	69.5 (2.57)	79.4 (2.26)
ResCNN-BGRU	76.4 (1.77)	89.2 (1.30)	**72.8** (2.15)	**86.4** (1.63)	**75.1** (2.47)	**84.1** (2.12)

The fold recognition accuracy (%) results are provided at the family, superfamily and fold levels, considering both the top 1 and top 5 ranked templates. We compare the CNN-GRU, CNN-BGRU, ResCNN-GRU and ResCNN-BGRU neural network models, trained with different loss functions: **(a)** Softmax loss with sigmoid activation, **(b)** LMCL with tanh activation, and **(c)** Thomson LMCL with tanh activation. Optimal LMCL hyperparameters are in Table 1. Boldface indicates the best performance per loss function. For each accuracy result, we also provide in parentheses the standard error estimated using 1000 bootstraps

The cross-validation fold classification accuracy on the training set for the different models and loss functions is shown in Fig. 3. When using softmax loss, we can observe that the models applying residual convolutions (ResCNN-GRU and ResCNN-BGRU) perform better at fold classification than their counterparts (CNN-GRU and CNN-BGRU). We also observe that the tanh activation function yields better results than the sigmoid activation for all tested margin values in the LMCL function. In this case, the scale value $s=30$ outperforms $s=50$ for both activation functions. As for the margin, larger values seem to further benefit models applying bidirectional GRU (CNN-BGRU and ResCNN-BGRU), suggesting that these models have a higher discriminative capacity. The optimal LMCL hyperparameters for each model are summarized in Table 1a.

In Table 2 we provide the fold recognition accuracy results on the LINDAHL test set (at the family, superfamily and fold levels), when using the cosine similarity (8) as ranking metric. Here, we used the optimal LMCL hyperparameters to train each model on the whole training set, from which we extracted the fold-related embeddings. Table 2a shows that the learned embeddings from the ResCNN-GRU and ResCNN-BGRU models using softmax loss yield slightly better fold recognition performance at the three levels than the CNN-GRU and CNN-BGRU models. On the other hand, in Table 2b we observe a large performance boost at all levels when introducing the LMCL as loss function in comparison with softmax loss. At the fold level, we achieve performance gains of 9 or more percentage points for most of the models. More precisely, the CNN-BGRU and ResCNN-BGRU models stand out for their remarkable results at the fold level, with 72.0% and 74.1% top 1 accuracy values respectively.

### Enhancing embedding discrimination power through Thomson-derived hyperspherical prototypes

We then tested the performance of our neural network models trained with a fixed matrix of prototypes $\mathbf {W} \in \mathbb {R}^{K\times d}$ in the classification layer (Fig. 1c), being the number of fold classes $K=1154$ and embedding dimension $d=512$. The fold prototype vectors have been maximally separated in the angular space by minimizing the THL–*sum* (6), using the $\mathbf {W}^{softmax}$ from each model as initial matrix (Fig. 1b). A detailed study comparing the performance of the two variants for the Thomson loss (THL–*sum* and THL–*maxcos*) and two options for the initial matrix ($\mathbf {W}^{softmax}$ and $\mathbf {W}^{random}$) can be found in Additional file 1: S2.

Given the optimized matrix of prototypes for each model, we tuned the LMCL scale and margin values by cross-validation over the SCOPe 2.06 training set, considering the tanh activation in the embedding layer. Results from this tuning are also shown in Fig. 3. As can be observed, the Thomson LMCL achieves better fold classification results, specially for the models applying residual convolutions, and particularly in the case of ResCNN-BGRU.

Finally, we set the optimal LMCL hyperparameters for each model (Table 1b) and trained them to extract fold-related hyperspherical embeddings. The fold recognition LINDAHL results in Table 2c show that, at the fold level, all the models benefit from the Thomson LMCL. Our best model, the ResCNN-BGRU, achieves top 1 accuracy values of 76.4%, 72.8% and 75.1% at the family, superfamily and fold levels, and top 5 accuracy of 89.2%, 86.4% and 84.1% at each level, respectively.

### Analysis of the hyperspherical embeddings

**Fig. 4:**
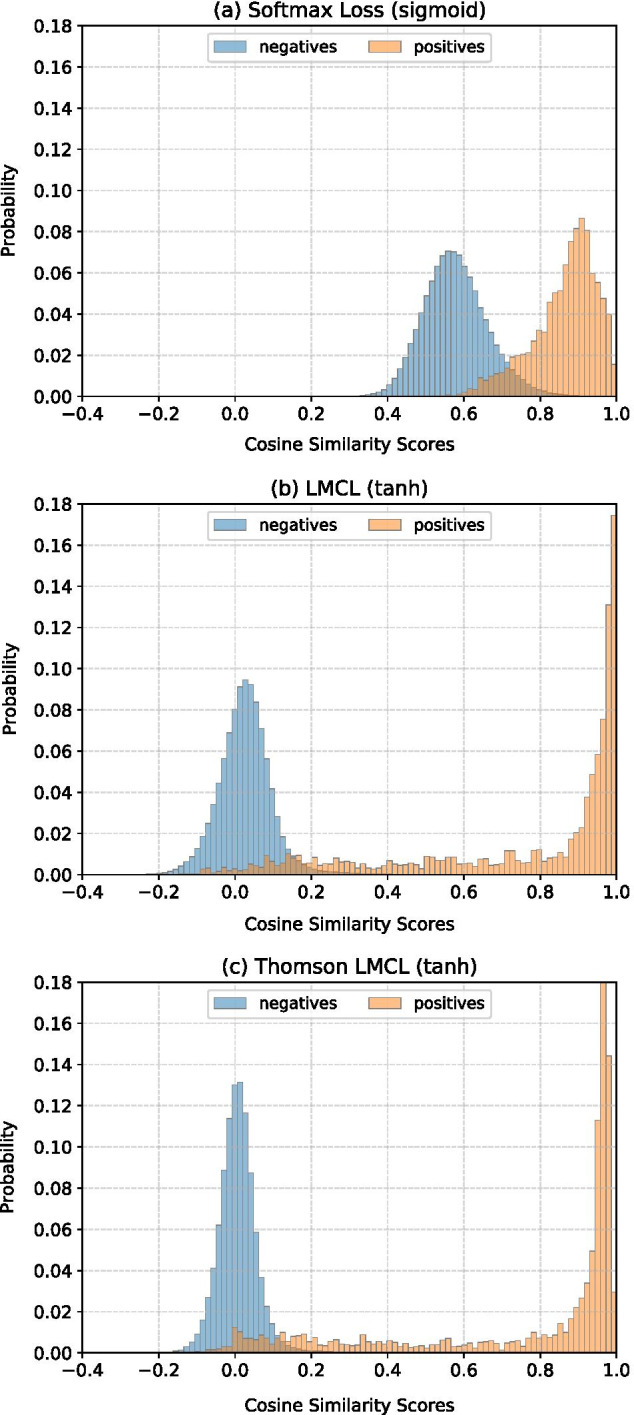
Cosine similarity probability histograms computed for all unique pairs within the LINDAHL test set (976 domains), grouping the negative pairs in blue color, and positive pairs in orange. To compute the cosine similarity scores, we used the embeddings extracted from the ResCNN-BGRU model trained with (**a**) softmax loss with sigmoid activation, (**b**) LMCL with tanh activation, or (**c**) Thomson LMCL with tanh activation

The fold recognition results of our FoldHSphere method using the ResCNN-BGRU model trained with hyperspherical prototypes reflect the effectiveness and discrimination capability of the learned hyperspherical embeddings. To further illustrate this, we analyzed the 512-dimensional embeddings extracted from the 976 protein domains in the LINDAHL dataset. Figure 4 compares the histogram of cosine similarities computed between each pair of embeddings for the softmax, LMCL and Thomson LMCL options. For each one, we plotted separately the histogram of negative pairs (different fold classes) and positive pairs (same fold class). It can be seen that the Thomson LMCL provides a better separation between positive and negative pairs, with a small overlap between the two groups. This directly contributes to a better performance in the pairwise fold recognition task. Additionally, we provide a two-dimensional visualization of the embedding space learned by the three loss functions in Additional file 1: Fig. S6, as well as a dendroheatmap of the hyperspherical embeddings obtained by the Thomson LMCL approach in Additional file 1: Fig. S7.

### FoldHSphere and FoldHSpherePro pairwise fold recognition performance results

Finally, we compare the results of our FoldHSphere and FoldHSpherePro approaches with several methods from the state-of-the-art, considering both the LINDAHL and LINDAHL_1.75 test sets. The FoldHSphere results correspond to those from the ResCNN-BGRU model trained with hyperspherical prototypes (Table 2). The FoldHSpherePro results were obtained after conducting a 10-stage cross-validation on a random forest model using the FoldHSphere scores along with other pre-computed protein pairwise similarities as inputs.

**Table 3:** Three-level LINDAHL fold recognition results of FoldHSphere and FoldHSpherePro in comparison with the state-of-the-art

Method	Family		Superfamily		Fold	
	Top 1	Top 5	Top 1	Top 5	Top 1	Top 5
PSI-BLAST [3]	71.2	72.3	27.4	27.9	4.0	4.7
HHpred [14]	82.9	87.1	58.0	70.0	25.2	39.4
RAPTOR [14]	**86.6**	89.3	56.3	69.0	38.2	58.7
BoostThreader [14]	86.5	90.5	66.1	76.4	42.6	57.4
SPARKS-X [15]	84.1	90.3	59.0	76.3	45.2	67.0
FOLDpro [34]	85.0	89.9	55.0	70.0	26.5	48.3
RF-Fold [34]	84.5	91.5	63.4	79.3	40.8	58.3
DN-Fold [34]	84.5	91.2	61.5	76.5	33.6	60.7
RFDN-Fold [34]	84.7	91.5	65.7	78.8	37.7	61.7
MRFalign [43]	85.2	90.8	72.4	80.9	38.6	56.7
CEthreader [48]	76.6	87.2	69.4	81.8	52.3	70.4
DeepFR (s2) [43]	65.4	83.4	51.4	67.1	56.1	70.1
DeepFRpro (s2) [43]	83.1	92.3	69.6	82.5	66.0	78.8
VGGfold [47]	67.9	84.3	53.2	68.4	58.3	73.5
CNN-BGRU [48]	71.0	87.7	60.1	77.2	58.3	78.8
CNN-BGRU-RF+ [48]	85.4	**93.5**	73.3	87.8	76.3	85.7
FoldHSphere	76.4	89.2	72.8	86.4	75.1	84.1
FoldHSpherePro	85.2	93.0	**79.0**	**89.2**	**81.3**	**90.3**

The accuracy (%) results are provided at the family, superfamily and fold levels, considering both the top 1 and top 5 ranked templates. Boldface indicates best performance

The three-level LINDAHL fold recognition results are shown in Table 3. We can see that our FoldHSphere method yields better top 1 accuracy values, above 12 percentage points at the superfamily and fold levels compared to the state-of-the-art method CNN-BGRU [48]. At the family level, we outperform all the deep learning methods. However, the alignment methods and approaches relying on pairwise similarities provide better results at this level. We include such information in the FoldHSpherePro method, which can be compared to DeepFRpro [43] and CNN-BGRU-RF+ [48] as all of them apply the same random forest ensemble approach. Our method provides a significant performance boost, obtaining remarkable top 1 accuracy results, with values of 79.0% at the superfamily level and 81.3% at the fold level. In terms of top 5 accuracy values, FoldHSpherePro also achieves the best performance, providing 89.2% and 90.3% at superfamily and fold levels respectively. On the other hand, at the family level we obtain on par results with the CNN-BGRU-RF+ method, being only outperformed by alignment and threading methods. This suggests that the performance of deep learning approaches might be saturating at this level. Similar conclusions can be drawn when evaluating the LINDAHL_1.75 test set (Table 4). Here we only compare to the DeepFR and CNN-BGRU methods, as they have been previously tested on such a dataset. The results show that our FoldHSpherePro approach also performs the best in this dataset, yielding top 1 accuracy values of 87.9%, 81.2% and 80.9% at the three levels respectively.

**Table 4:** Three-level LINDAHL_1.75 fold recognition results of FoldHSphere and FoldHSpherePro in comparison with the state-of-the-art

Method	Family		Superfamily		Fold	
	Top 1	Top 5	Top 1	Top 5	Top 1	Top 5
DeepFR (s2) [48]	72.2	85.6	46.6	64.3	50.8	67.1
DeepFRpro (s2) [48]	87.0	93.8	71.9	82.6	63.5	77.2
CNN-BGRU [48]	73.1	88.3	60.1	74.9	60.1	78.7
CNN-BGRU-RF+ [48]	**88.5**	**94.3**	74.0	86.3	71.1	86.8
FoldHSphere	77.9	89.0	72.4	87.0	75.8	84.3
FoldHSpherePro	87.9	94.1	**81.2**	**90.0**	**80.9**	**88.5**

The accuracy (%) results are provided at the family, superfamily and fold levels, considering both the top 1 and top 5 ranked templates. Boldface indicates best performance

**Fig. 5:**
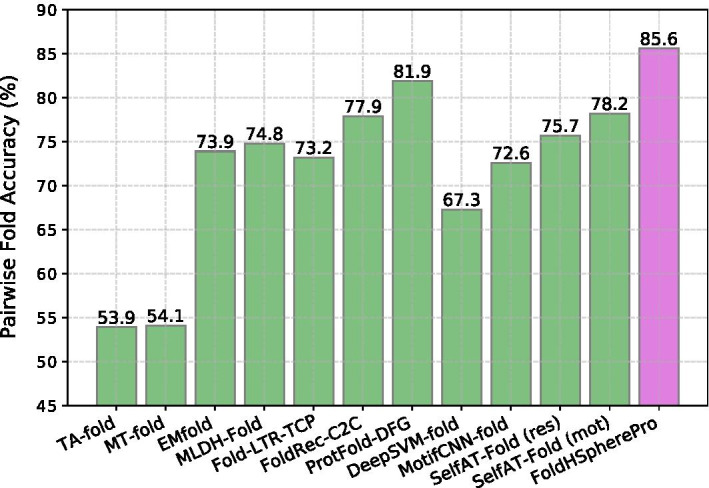
Fold-level LINDAHL fold recognition accuracy (%) results of our proposed FoldHSpherePro method in comparison with other ensemble methods from the state-of-the-art. The results are averaged over the two cross-validated subsets (LE_a and LE_b)

Figure 5 includes the evaluation results of the fold-level 2-stage cross-validation setting on the LINDAHL dataset (over subsets LE_a and LE_b). In this case, we only compare to ensemble methods that have been assessed with such a methodology, namely TA-fold [35], the multi-view learning frameworks MT-fold [36], EMfold [37] and MLDH-Fold [38], the learning to rank approaches Fold-LTR-TCP [39], FoldRec-C2C [40] and ProtFold-DFG [41], and the deep learning methods DeepSVM-fold [44], MotifCNN-fold [45] and SelfAT-Fold [46] (residue and motif options). The results in Fig. 5 show that our FoldHSpherePro method outperforms all of them yielding an accuracy of 85.6%.

## Discussion

In order to learn an embedding space that is representative of the protein folds, we have proposed a two-stage learning procedure. Our intuition here is that pre-defining the structure of the embedding space through fixed fold prototypes would ease the learning process for a neural network that embeds individual proteins into this space. The result of our experiments indicate that this intuition is well founded.

There are two important considerations when pre-defining the embedding space in our methodology: the initialization of the fold prototypes and how to obtain a suitable spatial distribution of these prototypes in the embedding space. Here we find that rather than initializing with random vectors in the hyperspherical space, better results are obtained by using the weight matrix from the classification layer of a neural network previously trained to map proteins to fold classes. Then, by maximally separating the weight vectors in this matrix in hyperspherical space, we obtain an effective configuration of the fold prototypes. We believe one of the main reasons is that such a matrix preserves the arrangement of the structural classes in the learned space, grouping related fold classes together and pushing them away from folds of unrelated structural classes (see Additional file 1: S2).

Our experimental results in Table 2 display a large performance boost at the superfamily and fold levels when comparing our methodology (using LMCL) to previous approaches that use the softmax loss. Our initial intuition for the lower performance of the state-of-the-art at these levels is that since evaluation is done for pairs of proteins, it is possible that two proteins from different folds lying near the fold classification boundary are closer to each other than they are to proteins from their respective folds. This informs our choice for using the LMCL as loss function, which introduces a margin between fold classes to avoid these cases.

A further performance gain is seen when combining the LMCL margin with the pre-trained fold prototypes (Table 2c). Here we use the fold prototypes optimized in the previous stage as a fixed (non-trainable) classification matrix for each neural network. We believe that the additional performance improvement is due to the simplified learning process that results from having this pre-defined organization of the folds in the embedding space, which is especially useful with limited and unbalanced training data. Stated differently, our models can focus on projecting protein embeddings closest to the corresponding fold prototypes without simultaneously learning where these prototypes should be.

We also observe from Fig. 3 and Table 2 that the models applying residual convolutions benefit more from the use of pre-trained prototypes compared to only optimizing with LMCL. This suggests the residual connections might extract more robust features for each amino acid, which seems to be helpful for the recurrent layer to obtain a better fixed-size representation for the whole protein domain. In particular, our ResCNN-BGRU architecture provides the best results, which can be attributed to its greater flexibility compared to the other tested architectures.

## Conclusion

In this work we have proposed the FoldHSphere method to tackle the protein fold recognition problem. We described a neural network training procedure to learn fold-representative hyperspherical embeddings for the protein domains. The embeddings were extracted from a residual-convolutional and recurrent network architecture (ResCNN-BGRU), which is trained by minimizing the angular large margin cosine loss (LMCL) around pre-defined prototypes for the fold classes. We used a Thomson-based loss function to maximally separate the fold prototypes in hyperspherical space. This way, our embeddings proved to be more effective at identifying the fold class of each protein domain by pairwise comparison. When evaluating the LINDAHL dataset, FoldHSphere alone provided a remarkable performance boost at the superfamily and fold levels, being competitive even with previous ensemble methods. Furthermore, our FoldHSpherePro ensemble method significantly improved the state-of-the-art results, outperforming the best method CNN-BGRU-RF+ at these levels. Therefore, due to their discrimination capability, the hyperspherical embeddings could be used to find template proteins even when the amino acid sequence similarities are low and thus advance in the template-based modeling of protein structures.

As future work, we will explore the application of recently proposed embeddings from language models pre-trained using millions of unannotated protein sequences for the protein fold recognition task, as they have shown promising results in several downstream tasks, such as protein secondary structure prediction and subcellular localization prediction [68–70].

## Supplementary Information

**Additional file 1.** Supplementary file 1.

## Data Availability

Source code, data and trained models can be found at http://sigmat.ugr.es/~amelia/FoldHSphere.

## Abbreviations

BGRU: Bidirectional gated-recurrent unit
CNN: Convolutional neural network
FC: Fully-connected
GRU: Gated-recurrent unit
LMCL: Large margin cosine loss
MSA: Multiple sequence alignment
NSL: Normalized softmax loss
PDB: Protein Data Bank
PSI-BLAST: Position-specific iterative basic local alignment search tool
PSSM: Position-specific scoring matrix
ResCNN: Residual-convolutional neural network
RF: Random forest
RNN: Recurrent neural network
SCOP: Structural Classification of Proteins
THL: Thomson loss
