# Supplementary material for: FoldHSphere: deep hyperspherical embeddings for protein fold recognition
Source: BMC Bioinformatics. 2021 Oct 12;22:490. doi: 10.1186/s12859-021-04419-7 (PMC8507389; doi:10.1186/s12859-021-04419-7)
Supplement: Supplementary file 1 — Additional file 1. Supplementary file 1. [file 12859_2021_4419_MOESM1_ESM.pdf]

# FoldHSphere: Deep Hyperspherical Embeddings for Protein Fold Recognition

Supplementary Material

Amelia Villegas-Morcillo, Victoria Sanchez, Angel M. Gomez

Department of Signal Theory, Telematics and Communications, University of Granada, Spain

## Contents

**1 Training Dataset and Cross-Validation Subsets** **2**

**2 Thomson-derived Hyperspherical Prototypes** **3**

2.1 Optimization curves . . . 3

2.2 Cosine similarity of prototype vectors . . . 3

2.3 Intra- and inter-structural class prototype separation . . . 4

2.4 Cross-validation performance and optimal set of prototypes . . . 5

**3 Effect of Secondary Structure Predictions on Performance** **7**

**4 Analysis of the Hyperspherical Embeddings** **8**

**5 Implementation Details** **9**

# 1 Training Dataset and Cross-Validation Subsets

**Table S1:** Number of protein domains, families, superfamilies and folds in each structural class within the SCOPe 2.06 training dataset.

| Structural class | Protein domains | Family classes | Superfamily classes | Fold classes |
|---|---|---|---|---|
| a | 2684 | 943 | 480 | 273 |
| b | 3603 | 858 | 343 | 168 |
| c | 4739 | 900 | 233 | 144 |
| d | 3882 | 1193 | 528 | 368 |
| e | 319 | 100 | 65 | 65 |
| f | 296 | 137 | 98 | 56 |
| g | 610 | 213 | 115 | 80 |
| Total | 16133 | 4344 | 1862 | 1154 |

**Table S2:** Cross-validation subsets for the SCOPe 2.06 training dataset. Here we split the 16133 protein domains into 5 groups, each one including domains from different family classes.

| Subset | Protein domains | | Family classes | | Superfamily classes | | Fold classes | |
|---|---|---|---|---|---|---|---|---|
| | Train | Valid | Train | Valid | Train | Valid | Train | Valid |
| CV1 | 12859 | 3274 | 3474 | 870 | 1641 | 615 | 1015 | 442 |
| CV2 | 12895 | 3238 | 3474 | 870 | 1642 | 606 | 1032 | 429 |
| CV3 | 12794 | 3339 | 3474 | 870 | 1633 | 630 | 1030 | 436 |
| CV4 | 13023 | 3110 | 3474 | 870 | 1646 | 611 | 1035 | 436 |
| CV5 | 12961 | 3172 | 3480 | 864 | 1626 | 632 | 1035 | 436 |

## 2 Thomson-derived Hyperspherical Prototypes

### 2.1 Optimization curves

In order to maximally separate our fold class prototypes in the hyperspherical space, we minimized a Thomson-related loss function to optimize the matrix $\mathbf{W} \in \mathbb{R}^{K \times d}$, being the number of fold classes $K = 1154$ and embedding dimension $d = 512$. We accelerated the optimization process by using the Adam optimizer [1] with a learning rate of $10^{-3}$. To check the convergence of the algorithm, at each iteration we monitored both the sum of all inverse distances (THL–*sum*) and the maximum cosine similarity between all pairs of prototypes. Figure S1 includes the optimization curves for the two variants, THL–*sum* or THL–*maxcos*, and initial matrices $\mathbf{W}^{softmax}$ from the CNN-GRU model or $\mathbf{W}^{random}$. When optimizing the THL–*sum* function, we see that the maximum cosine similarity between all pairs of prototypes decreases until a certain point in which starts increasing again. This suggests the algorithm is incorrectly trying to separate the majority of points, while bringing a few others together in order to further minimize the loss function. To avoid this unwanted behavior, we set the optimum iteration as the one with minimum value of maximum cosine similarity, which is 1130 for the $\mathbf{W}^{softmax}$ and 546 for the $\mathbf{W}^{random}$ initial matrices. On the other hand, the THL–*maxcos* function provides lower maximum cosine similarity, whereas the sum of inverse distances remains higher. In this case, we optimized for a huge number of iterations (50, 000).

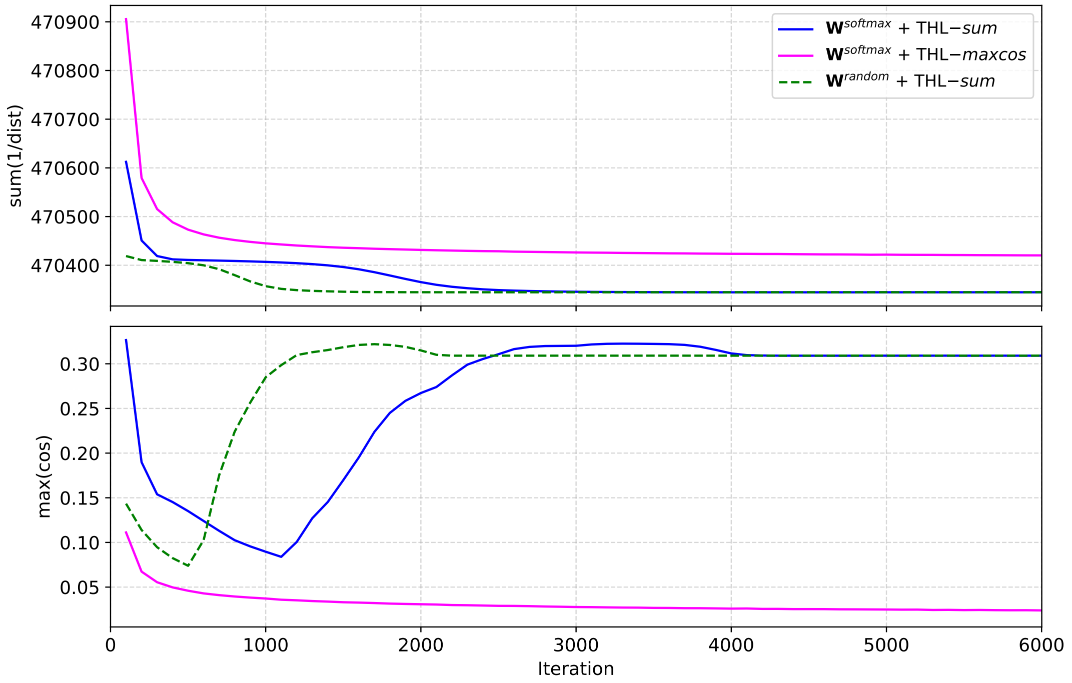


**Figure S1:** Thomson optimization curves at each iteration monitoring two metrics: sum of inverse of distances (above) and maximum cosine similarity (below) between all pairs of prototypes. For both metrics, we compare different options for initialization and loss function: initial matrix $\mathbf{W}^{softmax}$ and THL–*sum* (blue line), $\mathbf{W}^{softmax}$ and THL–*maxcos* (magenta line), or $\mathbf{W}^{random}$ and THL–*sum* (green dashed line).

### 2.2 Cosine similarity of prototype vectors

We then examined several structural characteristics from the optimized prototypes, in comparison with the initial matrices $\mathbf{W}^{softmax}$ and $\mathbf{W}^{random}$. In Figure S2, we plot the $K \times K$ cosine similarity matrix for each set of fold class vectors or prototypes, as well as the histogram of such pairwise cosine similarities. To ensure maximum angular separation between prototypes—given the number of points (fold classes) and dimensions in the hypersphere—the cosine values should be around 0 or negatives, as this translates into angles close to or greater than 90 degrees. We observe this in the cosine similarity histograms for the optimized prototypes. However, the cosine similarity matrix for the initial $\mathbf{W}^{softmax}$ suggests that the fold class vectors learned by softmax loss may contain rich information about the structural classes (i.e. 7 clusters can be observed, the same number as structural classes defined in SCOPe [2]).

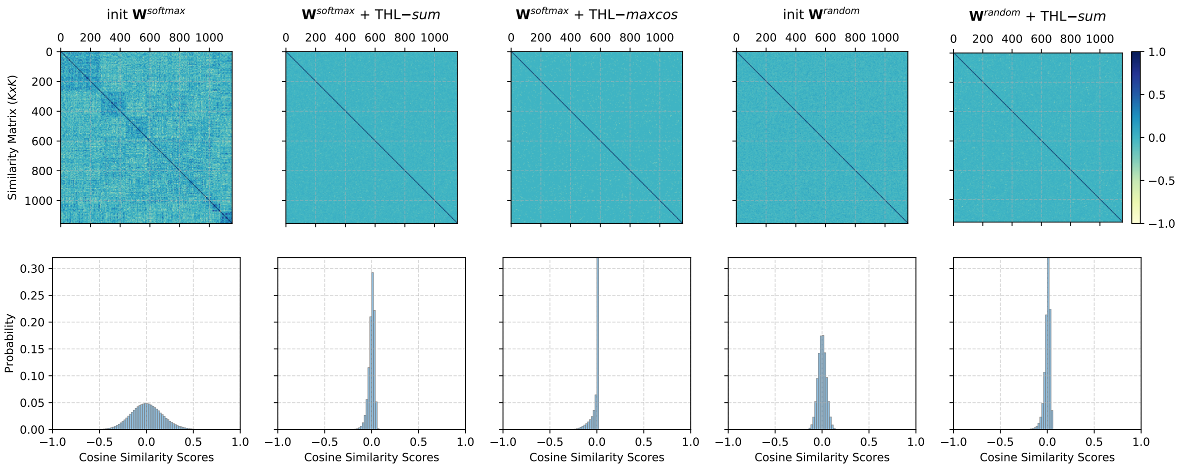


**Figure S2:** Cosine similarity matrices (above) and cosine similarity probability histograms (below) computed for the $K$ prototypes (corresponding to $K$ different folds). The compared sets of prototypes, from left to right are: initial matrix $\mathbf{W}^{softmax}$, optimized $\mathbf{W}^{softmax}$ with THL–*sum*, optimized $\mathbf{W}^{softmax}$ with THL–*maxcos*, initial $\mathbf{W}^{random}$, or optimized $\mathbf{W}^{random}$ with THL–*sum*.

## 2.3 Intra- and inter-structural class prototype separation

To evaluate the structural class information contained in the optimized prototypes, we grouped the $K$ fold prototypes into their respective structural classes (7 classes from $a$ to $g$ in SCOPe [2]). Then, we measured the average separation in terms of cosine distance, considering fold prototypes within the same structural class (intra-class separation), and prototypes from different structural classes (inter-class separation). We also computed the angular Fisher score (AFS) [3], defined as:

$$AFS = \frac{S_{inter}}{S_{intra}} = \frac{\sum_r \sum_{\mathbf{w}_j \in \mathbf{W}_r} (1 - \cos(\mathbf{w}_j, \mathbf{m}_r))}{\sum_r n_r (1 - \cos(\mathbf{m}_r, \mathbf{m}))}, \tag{1}$$

where $S_{inter}$ and $S_{intra}$ are the inter-class and intra-class scatter values, respectively. $\mathbf{W}_r$ is a subset of $\mathbf{W}$ containing $n_r$ prototypes from structural class $r$, $\mathbf{m}_r$ is the mean vector of those $n_r$ prototypes, and $\mathbf{m}$ is the mean vector of the whole set of prototypes.

In Figure S3, we can see that the optimized prototypes from the $\mathbf{W}^{softmax}$ using the THL–*sum* function retain the structural class information, with higher intra-class cosine similarity values than those across different structural classes. However, this information is not preserved when using $\mathbf{W}^{random}$ as initial matrix or the THL–*maxcos* as a loss function. Additionally, in Table S3 we observe that the initial $\mathbf{W}^{softmax}$ provides a lower angular Fisher score (AFS) value than the initial $\mathbf{W}^{random}$. Once more, this suggests that the prototypes in $\mathbf{W}^{softmax}$ are more informative about the structural classes. However, the AFS values decrease after optimizing both initial matrices with Thomson. This can be attributed to the significant increase in the cosine distance between all prototypes, which also increases the $S_{intra}$ term in equation (1). Overall, the $\mathbf{W}^{softmax}$ with THL–*sum* option provides a better angular Fisher score (0.9073) than the rest of options.

**Table S3:** Angular Fisher score of different sets of prototypes: initial matrix $\mathbf{W}^{softmax}$, optimized $\mathbf{W}^{softmax}$ with THL–*sum*, optimized $\mathbf{W}^{softmax}$ with THL–*maxcos*, initial $\mathbf{W}^{random}$, or optimized $\mathbf{W}^{random}$ with THL–*sum*. For the optimized sets of prototypes, we also include the selected iteration from the Thomson optimization algorithm.

| **Matrix** | initial $\mathbf{W}^{softmax}$ | $\mathbf{W}^{softmax}$ THL−*sum* | $\mathbf{W}^{softmax}$ THL−*maxcos* | initial $\mathbf{W}^{random}$ | $\mathbf{W}^{random}$ THL−*sum* |
|---|---|---|---|---|---|
| **Iteration** | — | 1130 | 50,000 | — | 546 |
| **AFS** | 0.9524 | 0.9073 | 0.9441 | 1.7031 | 0.9309 |

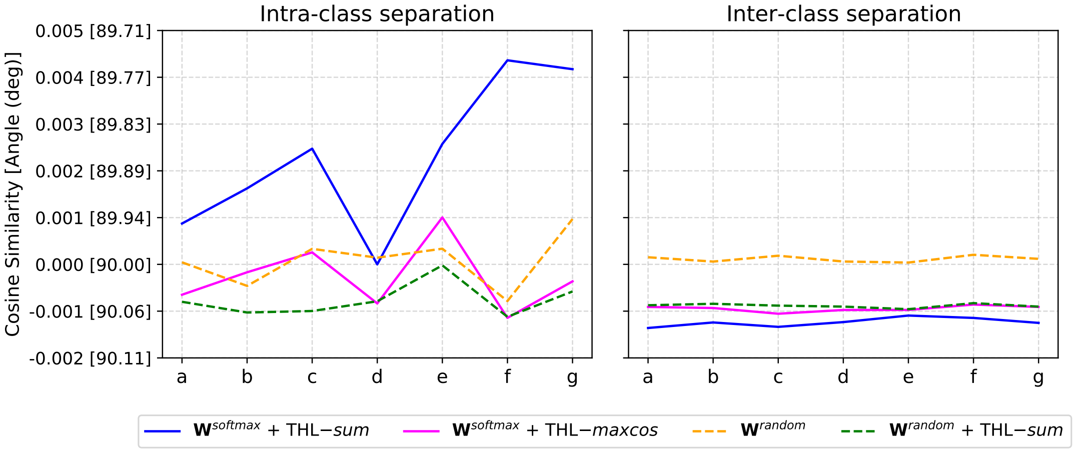


**Figure S3:** Intra- and inter-structural class separation of the $K$ prototypes (corresponding to $K$ different folds) considering the 7 structural classes $\{a, b, c, d, e, f, g\}$ defined in SCOPe [2]. The separation values have been measured in terms of cosine similarity and converted to angles in degrees. The intra-class separation (left) is computed for all pairs within the same structural class, while the inter-class separation (right) is computed for each prototype in one structural class with the rest of classes. Here, we compare different options for the set of prototypes: initial matrix $\mathbf{W}^{softmax}$ and THL–*sum* (blue line), $\mathbf{W}^{softmax}$ and THL–*maxcos* (magenta line), $\mathbf{W}^{random}$ before optimizing (yellow dashed line), or $\mathbf{W}^{random}$ and THL–*sum* (green dashed line).

## 2.4 Cross-validation performance and optimal set of prototypes

We then trained our neural network models CNN-GRU and ResCNN-GRU using the LMCL function and a fixed matrix of prototypes in the classification layer. In Figure S4 we compare the cross-validation performance of three optimized matrices by Thomson: $\mathbf{W}^{softmax}$ from each model with either THL–*sum* or THL–*maxcos*, and $\mathbf{W}^{random}$ with THL–*sum*. Here we applied the tanh activation in the embedding layer and used a scale $s = 30$ in the LMCL function. These results show that the set of prototypes derived from the $\mathbf{W}^{softmax}$ matrix with the THL–*sum* loss function yields a better fold classification performance than the other two options.

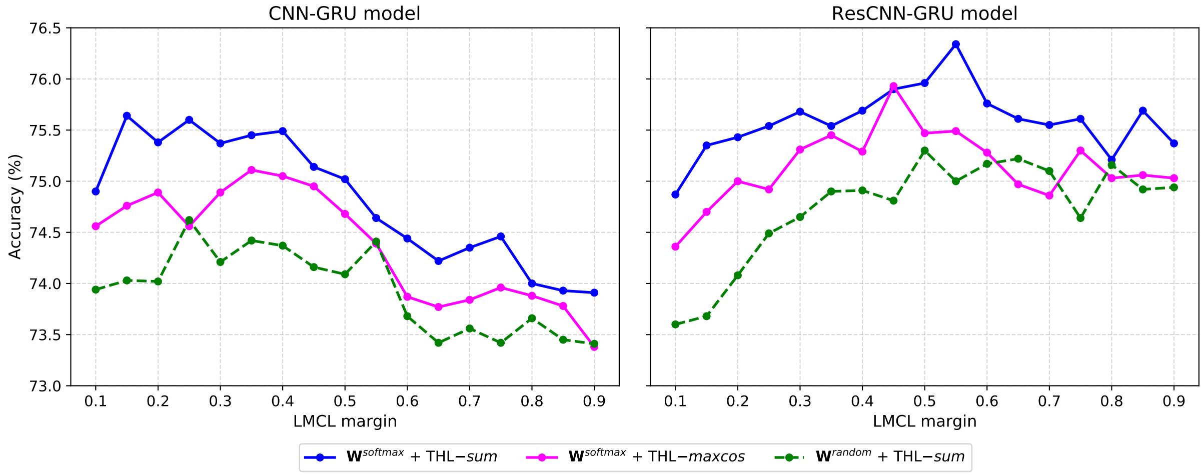


**Figure S4:** Cross-validation fold classification accuracy (%) results for different LMCL margins (with $s = 30$). The results are provided for the CNN-GRU and ResCNN-GRU models trained with Thomson LMCL. Here, we compare three options for the Thomson-optimized set of prototypes: initial matrix $\mathbf{W}^{softmax}$ and THL–*sum* (blue line), $\mathbf{W}^{softmax}$ and THL–*maxcos* (magenta line), or $\mathbf{W}^{random}$ and THL–*sum* (green dashed line).

Finally, we repeated the process for the rest of neural network models (considering their own matrix $\mathbf{W}^{softmax}$) and obtained the set of optimized prototypes by minimizing the THL–*sum*. The Thomson optimization curves and the optimal iteration for each model can be found in Figure S5. These optimized matrices are the ones we used to train the neural network models with the Thomson LMCL option.

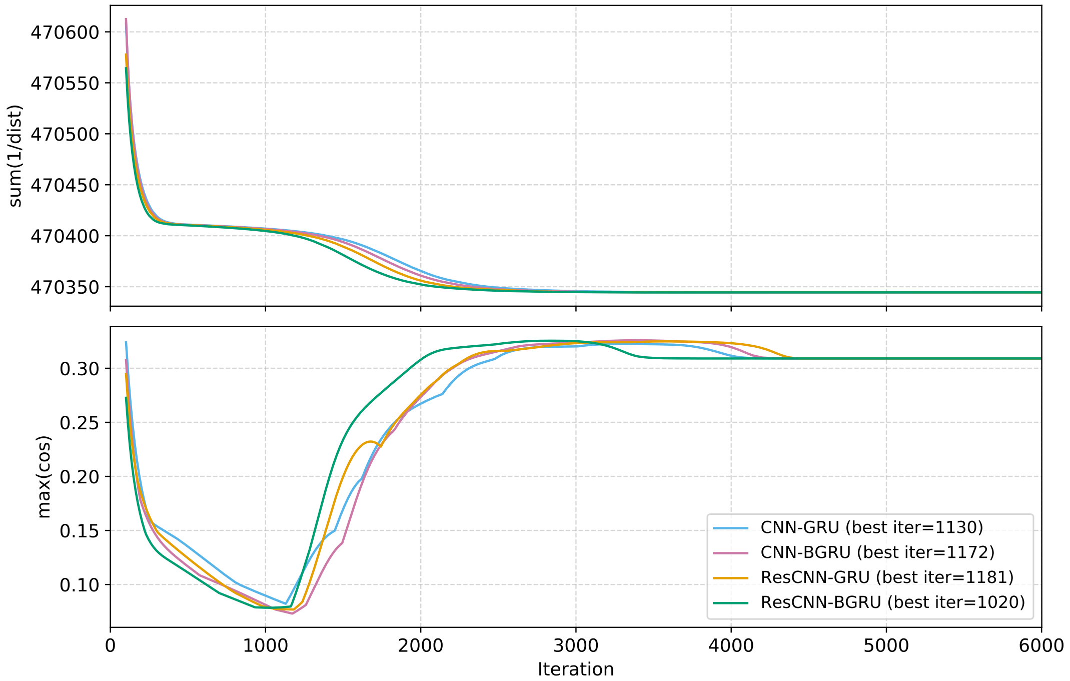


**Figure S5:** Thomson optimization curves at each iteration monitoring two metrics: sum of inverse of distances (above) and maximum cosine similarity (below) between all pairs of prototypes. We minimized the THL–*sum* considering $\mathbf{W}^{softmax}$ as initial matrix, trained from the models: CNN-GRU (soft blue line), the CNN-BGRU (soft pink line), the ResCNN-GRU (soft yellow line), or ResCNN-BGRU (soft green line).

# 3 Effect of Secondary Structure Predictions on Performance

In this work we represented the protein domain using a set of 45 features for each amino acid residue in the sequence, which include a one-hot encoding of the amino acid, the PSSM profile, as well as secondary structure and solvent accessibility predictions. As other methods from the bibliography [4, 5], we used the SSPro/ACCPro programs from SCRATCH-1D [6] to obtain predictions for the secondary structure and solvent accessibility. However, it must be noted that SSPro/ACCPro use homology analysis, so when the protein domain can be found in the PDB database they provide nearly perfect predictions of these features. In order to study the impact of using different predictors, we replaced our predictions with those given by SSPro/ACCPro "ab-initio" (i.e. without homology analysis) and NetSurfP-2.0 (hhblits) [7]. As the NetSurfP-2.0 method has been introduced quite recently, we expect it to provide better results than SSPro/ACCPro "ab-initio" and closer to the ones given by SSPro/ACCPro "homology".

Table S4 includes the results obtained at the test phase (using LINDAHL) by our ResCNN-GRU and ResCNN-BGRU models trained using either softmax loss, LMCL or Thomson LMCL. As we can see, a performance drop is shown when deactivating the homology analysis (SCRATCH-AB) but, in general, the proposed losses achieve better performance than softmax. NetSurfP-2.0, on the other hand, provides more similar results to those given by SCRATCH (homology), especially if we consider the top 5 accuracy results. However, it is difficult to draw conclusions without re-training our models using such predictions. Differences in performance could be explained by the fact that, in order to predict the fold class, some models might be more robust to secondary structure prediction errors than others.

**Table S4:** Effect of secondary structure and solvent accessibility predictions on FoldHSphere performance using the LINDAHL dataset. The fold recognition accuracy (%) results are provided at the family, superfamily and fold levels, considering both the top 1 and top 5 ranked templates. We compare the predictions given by SCRATCH (homology) [6], SCRATCH-AB (ab-initio) [6] and NetSurfP-2.0 (hhblits) [7] on the pre-trained ResCNN-GRU and ResCNN-BGRU neural network models, using different loss functions: **(a)** Softmax loss with sigmoid activation, **(b)** LMCL with tanh activation, and **(c)** Thomson LMCL with tanh activation.

| **Model** | **SS/ACC predictor** | **Family** | | **Superfamily** | | **Fold** | |
|---|---|---|---|---|---|---|---|
| | | **Top 1** | **Top 5** | **Top 1** | **Top 5** | **Top 1** | **Top 5** |
| **(a) Softmax Loss** | | | | | | | |
| ResCNN-GRU | SCRATCH | 72.6 | 90.3 | 59.4 | 77.0 | 58.9 | 75.1 |
| | SCRATCH-AB | 63.1 | 83.4 | 47.5 | 67.1 | 47.4 | 67.0 |
| | NetSurfP-2.0 | 72.1 | 88.8 | 56.5 | 74.4 | 62.6 | 75.7 |
| ResCNN-BGRU | SCRATCH | 76.8 | 91.2 | 65.0 | 82.0 | 59.5 | 76.6 |
| | SCRATCH-AB | 67.2 | 84.0 | 52.3 | 69.1 | 46.1 | 67.3 |
| | NetSurfP-2.0 | 73.9 | 87.4 | 56.7 | 75.3 | 55.1 | 74.1 |
| **(b) LMCL** | | | | | | | |
| ResCNN-GRU | SCRATCH | 75.7 | 89.7 | 66.4 | 81.1 | 67.6 | 80.1 |
| | SCRATCH-AB | 68.1 | 82.7 | 58.1 | 71.9 | 53.9 | 69.5 |
| | NetSurfP-2.0 | 74.2 | 89.5 | 62.0 | 77.4 | 70.4 | 78.8 |
| ResCNN-BGRU | SCRATCH | 75.1 | 89.5 | 69.8 | 85.3 | 74.1 | 82.2 |
| | SCRATCH-AB | 69.2 | 84.9 | 60.4 | 73.3 | 60.7 | 72.6 |
| | NetSurfP-2.0 | 75.1 | 90.1 | 64.3 | 84.1 | 70.4 | 80.1 |
| **(c) Thomson LMCL** | | | | | | | |
| ResCNN-GRU | SCRATCH | 76.9 | 89.5 | 69.1 | 82.9 | 69.5 | 79.4 |
| | SCRATCH-AB | 69.0 | 84.0 | 56.7 | 72.4 | 54.5 | 71.7 |
| | NetSurfP-2.0 | 78.0 | 89.2 | 65.4 | 81.8 | 69.5 | 78.8 |
| ResCNN-BGRU | SCRATCH | 76.4 | 89.2 | 72.8 | 86.4 | 75.1 | 84.1 |
| | SCRATCH-AB | 68.5 | 84.1 | 62.7 | 79.0 | 60.4 | 71.7 |
| | NetSurfP-2.0 | 75.5 | 88.3 | 66.8 | 83.6 | 67.3 | 81.0 |

# 4 Analysis of the Hyperspherical Embeddings

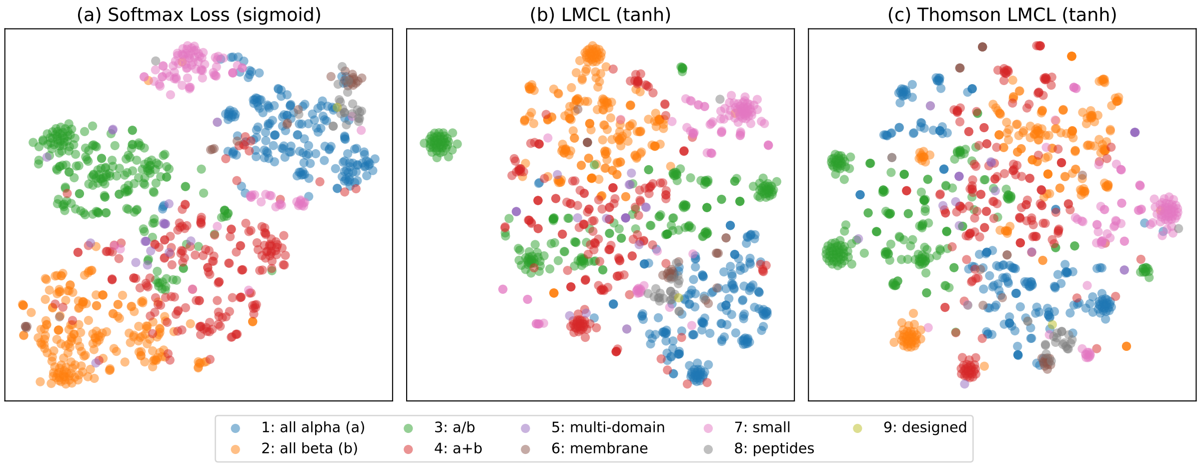


**Figure S6:** Visualization of the embedding space learned by the ResCNN-BGRU model trained with either (a) softmax loss with sigmoid activation, (b) LMCL with tanh activation, and (c) Thomson LMCL with tanh activation. Here, the 976 embeddings within the LINDAHL dataset have been projected into two dimensions by means of t-distributed stochastic neighbor embedding (t-SNE) [8], using a perplexity of 50 and 'cosine' as metric. The resulting points have been colored according to the structural class of each domain, which are named from 1 to 9 in SCOP 1.37 [9].

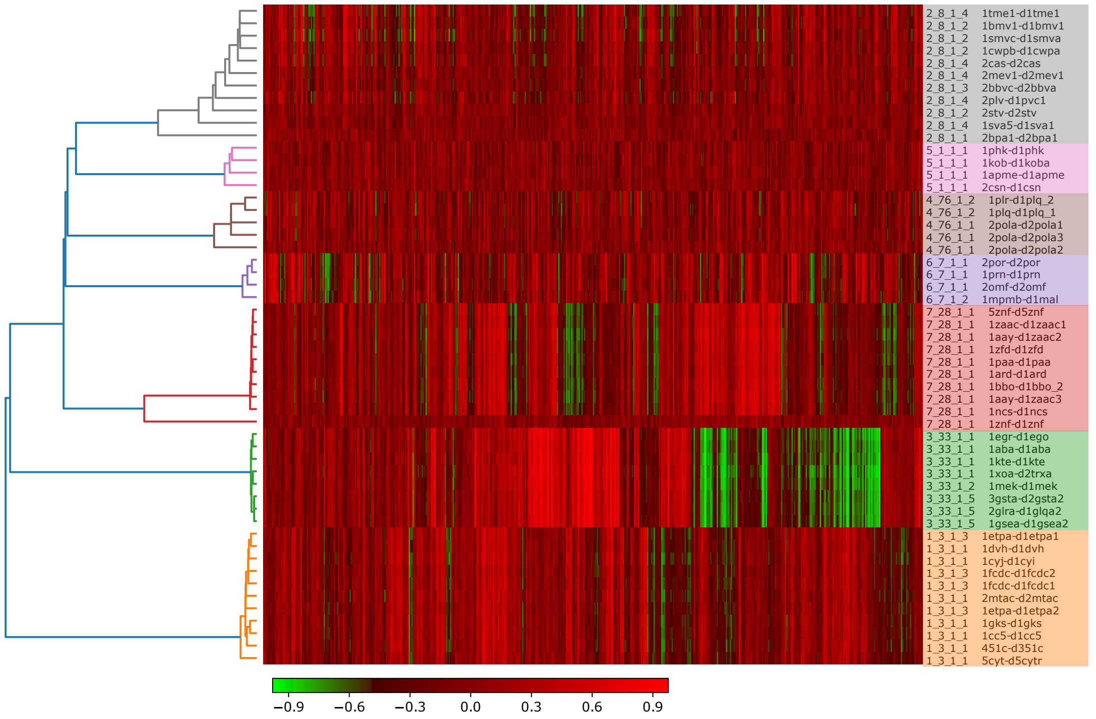


**Figure S7:** Dendroheatmap of the 512-dimensional hyperspherical embeddings extracted from the ResCNN-BGRU model trained with Thomson LMCL ($s = 30$ and $m = 0.6$). The analysis has been done by running bi-clustering over 53 protein domains from the LINDAHL test set, covering 7 folds named 1_3, 2_8, 3_33, 4_76, 5_1, 6_7 and 7_28. We computed the cosine distance between embedding vectors (rows) and embedding components (columns) separately. We then applied hierarchical clustering with single linkage to group similar vectors and components together. The individual elements in each embedding vector are colored according to their values (lower values in green and higher values in red). Note that the legend values range from $-1$ to 1, as the embeddings were extracted after applying the tanh activation function. We can see how the protein domains cluster together according to their embedding vectors into 7 differentiated clusters, one for each selected fold.

## 5 Implementation Details

We implemented our neural network models using Pytorch [10] (version 1.4.0) and Pytorch Lightning [11] (version 0.10.0), and were trained on a single GPU card (NVIDIA GTX Titan X, 12GB). The Thomson optimization algorithm was also implemented in Pytorch and run on a single GPU card. On the other hand, we used the Python Scikit-learn [12] package (version 0.23.1) implementations to train the random forest models and visualize the embedding space by means of t-SNE. Source code and data needed to reproduce the results of this paper can be found in `http://sigmat.ugr.es/~amelia/FoldHSphere`.

## References


[1] Diederik P. Kingma and Jimmy Ba. "Adam: A method for stochastic optimization". In: *arXiv preprint arXiv:1412.6980* (2014).

[2] Naomi K. Fox, Steven E. Brenner, and John-Marc Chandonia. "SCOPe: Structural Classification of Proteins–extended, integrating SCOP and ASTRAL data and classification of new structures". In: *Nucleic Acids Research* 42.D1 (2014), pp. D304–D309.

[3] Weiyang Liu, Yandong Wen, Zhiding Yu, et al. "SphereFace: Deep Hypersphere Embedding for Face Recognition". In: *Proceedings of the IEEE Conference on Computer Vision and Pattern Recognition (CVPR).* 2017, pp. 212–220.

[4] Jie Hou, Badri Adhikari, and Jianlin Cheng. "DeepSF: deep convolutional neural network for mapping protein sequences to folds". In: *Bioinformatics* 34.8 (2018), pp. 1295–1303.

[5] Amelia Villegas-Morcillo, Angel M. Gomez, Juan A. Morales-Cordovilla, et al. "Protein Fold Recognition from Sequences using Convolutional and Recurrent Neural Networks". In: *IEEE/ACM Transactions on Computational Biology and Bioinformatics* (2020).

[6] Christophe N. Magnan and Pierre Baldi. "SSpro/ACCpro 5: almost perfect prediction of protein secondary structure and relative solvent accessibility using profiles, machine learning and structural similarity". In: *Bioinformatics* 30.18 (2014), pp. 2592–2597.

[7] Michael S. Klausen, Martin C. Jespersen, Henrik Nielsen, et al. "NetSurfP-2.0: Improved prediction of protein structural features by integrated deep learning". In: *Proteins: Structure, Function, and Bioinformatics* 87.6 (2019), pp. 520–527.

[8] Laurens Van der Maaten and Geoffrey Hinton. "Visualizing data using t-SNE". In: *Journal of Machine Learning Research* 9.11 (2008), pp. 2579–2605.

[9] Alexey G. Murzin, Steven E. Brenner, Tim Hubbard, et al. "SCOP: A structural classification of proteins database for the investigation of sequences and structures". In: *journal of Molecular Biology* 247.4 (1995), pp. 536–540.

[10] Adam Paszke, Sam Gross, Soumith Chintala, et al. "Automatic differentiation in pytorch". In: (2017).

[11] William A. Falcon et al. "PyTorch Lightning". In: (2019). URL: `https://github.com/PyTorchLightning/pytorch-lightning`.

[12] Fabian Pedregosa, Gaël Varoquaux, Alexandre Gramfort, et al. "Scikit-learn: machine learning in Python". In: *journal of Machine Learning Research* 12 (2011), pp. 2825–2830.
